# Progress and criticalities in the management of acute promyelocytic leukemia

**DOI:** 10.18632/oncotarget.22385

**Published:** 2017-11-12

**Authors:** Francesco Lo-Coco, Laura Cicconi, Maria Teresa Voso

**Affiliations:** Francesco Lo-Coco: Department of Biomedicine and prevention, University Tor Vergata, Rome, Italy

**Keywords:** PML/RARA, arsenic trioxide, ATRA, early death, differentiation syndrome

In only few decades, remarkable advances in biology and therapy have transformed acute promyelocytic leukemia (APL), once regarded as the most rapidly fatal human leukemia, into a paradigm of targeted treatment in human cancer, with most patients being nowadays curable without any or with only small amounts of conventional chemotherapy. Yet, the management of this rare subtype of leukemia remains today highly challenging, due to its aggressive presenting features and to some peculiar disease- and treatment-related complications.

**Figure 1:**
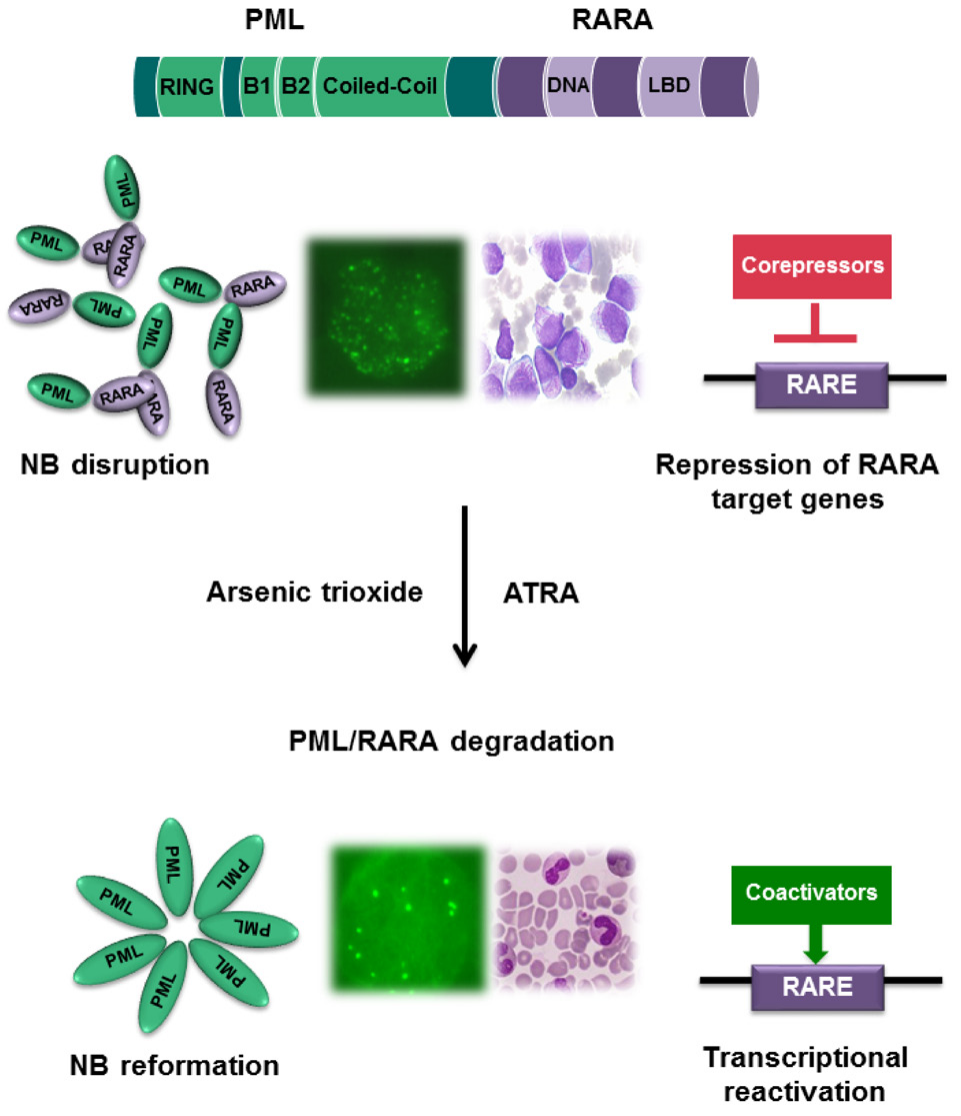
Mechanism of action of ATRA and ATO in APL The PML/RARA oncoprotein induces block of myeloid differentiation and blast proliferation through i) binding at high affinity to the corepressor complex with consequent transcription repression of a number of RARA target genes; ii) PML/RARA disrupts PML nuclear bodies structure (NB). The combination of ATRA and arsenic trioxide (ATO) induces the degradation of the PML/RARA oncoprotein through the ubiquitin-proteasome pathway. PML/RARA promoter clearance induces transcriptional reactivation of target genes and nuclear bodies reformation, which ultimately induces terminal differentiation of leukemic cells.

The APL-unique t(15;17) translocation, which generates the PML/RARA oncoprotein is the key pathogenetic event of APL. This hybrid protein results in the differentiation block of bone marrow myeloid precursors and is responsible for the exquisite sensitivity of the disease to all-trans-retinoic acid (ATRA) and arsenic trioxide (ATO). Mechanistically, PML/RARA acts as an aberrant retinoic acid receptor alpha (RARA) and displays an increased affinity for the chromatin remodeling and methylating enzymes leading to transcriptional repression of genes critical for myeloid differentiation. ATRA induces terminal differentiation of leukemic promyelocytes by binding RARA and inducing the transcriptional re-activation of target genes repressed by PML/RARA as well as the direct degradation of the oncoprotein. ATO in turn targets PML/RARA by binding to its PML moiety and induces the degradation of the oncoprotein, thus restoring the formation of matrix-associated nuclear bodies, finally resulting in APL cell death (Figure 1) [1].

Due to the severe bleeding diathesis frequently present at diagnosis, APL remains one of the most aggressive forms of leukemia and has to be managed as a medical emergency. Institution of supportive care (i.e. platelets, fibrinogen and/or fresh frozen plasma transfusions) together with prompt initiation of ATRA must immediately follow the clinical suspect of APL [2]. However, genetic diagnostic confirmation to identify PML/RARA is mandatory and can be readily obtained by FISH or PCR testing in leukemic blood or (preferably) bone marrow cells. The bleeding tendency due to an imbalance between procoagulant, anticoagulant, and fibrinolytic activities is triggered by circulating APL cells and can cause lethal hemorrhagic events within the first hours or days from diagnosis or sometimes even before APL diagnosis is suspected. Differentiation induced by ATRA results in the loss of procoagulant and fibrinolytic properties of APL cells, with improvement of the hypercoagulable state [3].

APL is classified as low/intermediate- or high- risk according to the platelet (PLT) and white blood cell (WBC) counts at the time of initial diagnosis (i.e. PLT < or > 40x10^9^/L, and WBC > 10x10^9^/L, respectively). This classification reflects disease severity, but it is not clear whether high-risk APL represents an advanced disease phase or a distinct biological disease subset. Until recently, ATRA combined to anthracyclines has been the standard of care for newly diagnosed patients. [2] For low/intermediate risk APL, a chemotherapy-free regimen combining ATO and ATRA has been shown to be superior compared to ATRA and chemotherapy and has become the new standard. In fact, two large randomized studies comparing ATRA-ATO vs. ATRA and chemotherapy showed significantly better outcomes for the chemo-free option together with inferior toxicity both in terms of short-term (i. e. cytopenias, infections, mucositis) and long-term (i.e. therapy-related myeloid disorders) complications [4, 5].

It is highly recommended that patients with APL be treated in specialized hematology centers. The most critical period remains the induction phase characterized by possible thrombo-hemorrhagic events, cytopenias and infectious complications, and particularly by the frequent occurrence of a differentiation syndrome (DS). Originally referred to as “retinoic-acid syndrome”, DS is a relatively common complication (approximately 20% of patients develop it) that derives from the induction of myeloid cell differentiation and from a general inflammatory state. Signs and symptoms include unexplained fever, weight gain, dyspnea with pulmonary infiltrates, pleuro-pericardial effusion, hypotension, and renal failure [6]. Initially reported as associated to ATRA only, DS was later described to occur with the use of other drugs such as ATO in APL and, more recently, AG-221 in the treatment of IDH2-positive AML [7]. In APL, DS may occur in the first week of therapy of ATRA and/or ATO and ATRA-chemotherapy or later on, during the second and particularly the third week. Its prompt recognition and early initiation of high-dose steroids treatment is recommended due to its life-saving potential while the benefit of steroids used in prophylaxis is unclear. The prevalence of DS does not seem to be different when using ATRA-ATO or ATRA-chemotherapy based regimens [6].

After the induction phase, current treatment options include at least 2-3 cycles of consolidation (with ATRA-ATO or with ATRA and chemotherapy, based on the initial treatment option) whereas there is no consensus on the use of maintenance whose benefit has remained questionable. One very important treatment objective in APL is the achievement of molecular remission (MR, i.e. a PCR-negative status for PML/RARA) at the end of consolidation, while the cost-effectiveness of prolonged monitoring after the achievement of MR is controversial in light of the very high probabilities of cure with modern regimens.

Among future challenges, a better understanding and control of the coagulopathy is warranted, as early hemorrhagic death remains the most frequent cause of failure. Furthermore, clinical studies should focus on the role of ATO in high risk, elderly and pediatric patients. Finally, the implementation of chemo-free regimens by the inclusion of oral formulations of arsenic may further improve the tolerability of this highly curative treatment, particularly in terms of patient quality of life.
